# Integrating topics of sex and gender into medical curricula—lessons from the international community

**DOI:** 10.1186/s13293-016-0093-7

**Published:** 2016-10-14

**Authors:** Virginia M. Miller, Georgios Kararigas, Ute Seeland, Vera Regitz-Zagrosek, Karolina Kublickiene, Gillian Einstein, Robert Casanova, Marianne J. Legato

**Affiliations:** 1 Departments of Surgery and Physiology and Biomedical Engineering, Mayo Clinic, 200 First St. SW, Rochester, MN 55905 USA; 2 Institute of Gender in Medicine and Center for Cardiovascular Research, Charite University Hospital and DZHK (German Centre for Cardiovascular Research) Partner Site, Berlin, Germany; 3 Centre for Gender Medicine, Karolinska Institutet, Stockholm, Sweden; 4 Department of Psychology, University of Toronto, Ontario, Canada; 5 Texas Tech University Health Science Center, Lubbock, TX USA; 6 Columbia University, New York, NY USA

**Keywords:** Educational platforms, Web-based, Resources

## Abstract

In the era of individualized medicine, training future scientists and health-care providers in the principles of sex- and gender-based differences in health and disease is critical in order to optimize patient care. International successes to incorporate these concepts into medical curricula can provide a template for others to follow. Methodologies and resources are provided that can be adopted and adapted to specific needs of other institutions and learning situations.

## Background

Biomedical advances into the human genome have brought personalized or individualized medical care to the forefront. In addition, political and social forces initiated by laywomen to expand the definition of women’s health beyond the breast and reproduction propelled the need for a more comprehensive characterization of women’s unique physiology. Subsequently, the science of gender-specific medicine developed. This discipline is now encompassing a larger view comparing both sexes not only in humans but at all levels of biomedical research. Incorporating and translating new discoveries in sex and gender research into programs to train the next generation of scientists and health-care providers is challenging but provides opportunities to improve and modernize educational approaches, which will ultimately result in improved patient care.

Several efforts have been initiated around the world to assure continued research into sex and gender differences. In particular, in 2010, the Canadian Institutes of Health required the discussion of the inclusion of sex or gender in both non-human animals and humans in grant applications. In addition, there is a 2020 European Union Strategic Vision for Gender (http://ec.europa.eu/research/participants/data/ref/h2020/grants_manual/hi/gender/h2020-hi-guide-gender_en.pdf), and in 2015, the United States National Institutes of Health developed policies (NOT-OD-15-102; NOT-OD-15-103) requiring investigators to account for sex as a biological variable in preclinical research. These policies and plans affirm the legitimacy and importance of sex- and gender-based or gender-specific science.

Programs to incorporate new knowledge of sex and gender differences into various venues for training the next generation of scientists and health-care providers are also emerging. In October 2015, a summit was held at Mayo Clinic in Rochester, MN (http://sgbmeducationsummit.com/summit-proceedings/), in which a group of international scientists and educators from prominent universities participated in a panel to discuss their experiences in developing and implementing these training programs. Although separated by distance and culture, several common themes emerged in their efforts to embed concepts of sex and gender into medical education.

One common theme emerging from the panel discussion that challenges the progress of bringing concepts of sex and gender into the medical curricula was the preconceived and unconscious biases surrounding the terms sex- and gender-based medicine. These misunderstandings and biases limit the understanding of the benefits and application of the concepts to overall patient care. Therefore, an essential requirement for developing educational platforms is a clear articulation of what sex and gender mean. “Sex” is a biological variable referring to differences defined by sex chromosomes (XX, XY) and the presence of functional reproductive organs, whereas “gender” is a cultural construct referring to behaviors thought to be specified by psychosocial expectations that accrue on the basis of perceived or assigned sex [1]. The Canadian Institute of Health Research defines gender as the socially constructed roles, behaviors, expressions, and identities of an individual. Gender influences how people perceive themselves and each other, how they act and interact, and the distribution of power and resources in society (http://www.cihr-irsc.gc.ca/e/47830.html). Gender, as defined by the Gendered Innovations group, is the constellation of sociocultural processes that interact with and influence human biology (https://genderedinnovations.stanford.edu/what-is-gendered-innovations.html). This group expands the definition of gender by clarifying how individuals and groups perceive and present themselves as “gender identity,” spoken and unspoken rules—in family, workplace, institutional, or global culture—that influence individual attitudes and behaviors as “gender norms,” and the power relations between individuals of different gender identities, such as the relations between a patient and physician as “gender relations.” That is, gender represents the total of the social and cultural variables by which an individual’s environment through nurture (culture) influences nature (biology) or how the environment “writes” on the body [2]. Thus, as our understanding develops regarding epigenetic modification of the genome by the environment, age, and hormonal status, it is evident that sex and gender are a composite. To separate out the contribution of chromosomes and the milieu in which the person exists/has existed is virtually impossible. For humans, a separate consideration of “sex” and “gender” is artificial and, indeed, may be impossible to implement accurately in some situations.

The second common theme emerging from the panel discussion was that in order to implement change in the curricula, an individual or a key group of individuals must act as the “change agent(s) championing the vision. Unfortunately, most often, the change agent(s) was self-designated by vision and passion. However, for success, financial and structural resources need to be committed from the highest level of governmental or institutional leadership in order for the vision to be realized. A summary of each panel participant’s processes and successes is provided so that others can facilitate change and implement similar programs at their respective universities.

## Germany

In Berlin (Germany), the Institute of Gender in Medicine (GiM), Charite University Hospital, undertook a systematic approach to incorporate sex and gender medicine into medical and inter-professional education. Their integrative approach consisted mainly of three components.

The earliest and most basic component was the development of an open-access database of gender medicine. The GenderMed database (http://gendermeddb.charite.de) was established through the systematic collection of literature on gender-related issues across ten major medical disciplines in a structured manner, including cardiology, endocrinology, and neurology [3]. The database was made possible by two grants from the German Ministry of Education and Research (BMBF, 2008–2010 and 2012–2014). It includes more than 11,000 publications that deal with sex and gender in medicine and research, thereby offering a comprehensive overview of sex and gender medicine and research. In particular, the GenderMed database is a free user-friendly tool that is crucial for the retrieval, analysis, and archiving of literature specific to gender medicine, and it can also be used as a training tool in medical schools, postgraduate programs, and vocational training of doctors and researchers.

The most recent and most important component of the systematic approach was the integration of sex and gender aspects into the new modular medical curriculum of the medical school, a process that started in 2010 [4]. This integration was accomplished by close collaboration with the Department for Course Development, where a change agent for gender medicine was placed in the Curriculum Development Team [4]. This change agent was crucial for the communication and interaction with the dean, professors of different departments, gender researchers and experts, and the GiM, among others. The overall goal was the incorporation of the aspects of sex and gender differences in the development, diagnosis, and therapy of disease into the new modular medical curriculum for the teaching of medical students. These efforts, which were based on the database described above, resulted in 94 lectures, 33 seminars, and 16 practical courses on gender medicine and sex-related research throughout the modular curriculum [4]. The GiM also organized and offers a whole module on “gender-specific diseases.”

The final component of the integration was the creation of a module for postgraduate students, as well as other professionals. In particular, this module is an interactive, web-based eLearning platform on gender medicine (http://egender.charite.de). The GiM developed this advanced training course based on the systematic analysis of sex and gender differences in preclinical and clinical research (Seeland et al., submitted) in a European teaching context made possible by an EU grant (EUGIM, 2009–2011), coordinated by GiM. The overall aim of this eLearning course is to increase the participant’s knowledge on sex differences and gender aspects in medicine and research. It offers a platform for the discussion of major gaps in gender medicine and sex-related research, thereby supporting the growth of the community of gender medicine. The eGender Medicine course can be employed for the teaching of medical students, postgraduate students in different modular master’s programs, and for the vocational training of doctors and researchers.

Collectively, this systematic and integrative approach with these three main components has led to the successful incorporation of sex and gender medicine into medical and inter-professional education both locally and worldwide through the eLearning solutions. These programs contribute to the development and growth of the gender medicine discipline and community.

## Sweden

The Center for Gender Medicine (CfGM) at Karolinska Institutet (KI) was the first in Europe to establish the web-based educational course Health and Disease from a Gender Perspective. This course was initially given as a freestanding course for undergraduate medical education and currently is offered as an elective and flexible course at KI. The target groups are those with a major (at least 60 high school points or credits) in health care, life sciences, biotechnology, bio-entrepreneurship, and master students in the health-care sciences. As such, it is virtually open to all students at KI and other Swedish universities. As the course is available both in Swedish and in English, it also acts as a platform for international collaboration. For example, the course is being utilized by students from Lodz Medical University (Poland). Overall, the course encourages students to examine the validity and implications of the statement “Every cell has sex and every person is gendered” [1]. In addition, it focuses on what current research reports regarding the social, biological, and psychological similarities and differences between the sexes and genders, as well as discussion of their ramifications for illness, health, rehabilitation, and health care for women, men, and gender-diverse people. One advantage of this course is that it could be used as a “fast track” course during the first 10 weeks of the autumn/spring semester or could be modified accordingly to the program into which students are enrolled.

The web-based elective course represents a major component of the currently available online learning platform. It is combined with in-house learning, which is creative, flexible, and accessible to many people and aims to attract students from a wide variety of professional backgrounds, including those working clinically as well as those interested in a career in basic research. The strength of the platform is that it incorporates other online tools, such as “Gendered Innovations” (www.genderedinnovations.se) and the “International Women’s Health and Human Rights” open online course, developed by Stanford University and adjusted at KI. In addition, a number of in-house seminars with guest lecturers (Meet an Expert—Get Inspired) facilitate and enhance the learning process as it draws on team-based learning approaches while promoting a sense of community among the students.

According to KI regulations, the undergraduate education should be strongly endorsed by doctoral education, and the 1-week doctoral course “SEX and Gender Aspects in Biomedical Research” is available for PhD students and postdoctoral fellows with an ultimate goal to learn how to make research gender sensitive (e.g., http://ki.se/sites/default/files/programpostgraduate_kurs2013.pdf).

The CfGM is also continuously engaging an international team of students, managers, and engineers to develop interdisciplinary teaching resources and case studies for undergraduate and graduate programs (http://ki.se/en/research/education-at-centre-for-gender-medicine).

An educational platform currently under an implementation phase at KI is called “Flexibel kommUnikativ inTerprofessionell Utbildnings och implementeRingsplattform för gEnusmedicinska aspekter för hälso- och sjukvård (*FUTURE*).” The FUTURE educational platform is a web-based environment for teaching that acts as a complement to traditional teaching at different undergraduate programs. The crucial element is currently existing but continuously updated and re-named as web-based elective course “Future Challenges for Health Care: Gender Adds Value.” This elective course consists of three modules, including basic understanding, enhancement, and implementation together with physical meetings between those studying at different health-care and biomedicine programs. The platform also aims to include direct communications with teachers via a specially designed course “Putting Gender on the agenda.” This platform is designed for mentors/teachers at KI and Karolinska University Hospital in order to strengthen reflections, communications, and development of novel mindset for all stakeholders in academia and health care. The FUTURE platform includes the open knowledge database developed by Stanford University and adjusted for the Swedish audience (www.genderedinnovations.se). This knowledge database presents an overview of important topics and cases where implementation of sex and gender perspectives leads to innovation. Moreover, the Swedish platform of “*genderedinnovations*” acts as an umbrella to promote and utilize other international teaching resources developed by other countries (i.e., Canadian Institute of Gender Health, GiM, Mayo Clinic, and TTUHSC). The Stockholm County Council considers this a potential learning platform for so-called specialized education for health-care providers, as well as for educational activities towards patient’s organization, society in general, and industry and commerce.

## Canada

Through its national funding body, the Canadian Institutes of Health Research and its Institute of Gender and Health (IGH), as well as through the University of Toronto’s Collaborative Graduate Program in Women’s Health (CGPWH), Canada has been building capacity in sex and gender for research trainees in order to advance knowledge in how to provide validated evidence of sex- and gender-based differences in pathophysiology and human health. These trainees include medical students and physicians, but the program to date is principally geared towards graduate students. Focusing on multidisciplinary training in sex and gender, across disciplines as diverse as immunology, medical sciences, anthropology, religion, and public health (to mention only 5 of 13 collaborating units), the CGPWH aims to instill an appreciation for both the social and the biological in health conditions for both women and men. From 2011 to 2013, IGH sponsored a 1-week summer institute for graduate students and postdoctoral fellows with faculty mentors in both qualitative and quantitative methodologies covering sex differences, embodiment, and masculinities, leading to lasting student engagement in studying sex together with gender. IGH also designed online training through webinars (http://www.cihr-irsc.gc.ca/e/48641.html), a research case book (http://www.cihr-irsc.gc.ca/e/44082.html), and training modules in sex and gender for researchers, grant reviewers, and students (https://www.youtube.com/watch?v=fdftL6S94hs). None of these initiatives are without their costs. The Institute of Gender and Health at the Canadian Institutes of Health Research has its own operating budget which, in the past, allowed it to launch its own sex and gender initiatives and now includes more and more cooperation of the other 12 institutes. The Collaborative Graduate Program in Women’s Health operates on a minimal budget with a dedicated executive committee and some small administrative support—echoing the point that most of these programs require the dedication of a champion to keep them running. There is no information about the impact of these programs beyond the academic setting, but at least in the academic setting, there is a growing awareness of the importance of considering sex and gender. While there is some medicine-specific training at individual medical schools (e.g., Ottawa), it is not yet a national effort. However, lessons learned from integrating sex and gender through graduate training can now be well-applied to medical education.

## United States of America

In the USA, Texas Tech University Health Sciences Center (TTUHSC) started from the ground up with a needs assessment led by a collaborative group of change agents incorporating multiple stakeholders including education deans, medical faculty, preclinical faculty, inter-professional faculty, and most importantly, medical students. The final product was a complete mapping of the sex and gender content within the basic science medical curriculum performed by student auditors who were paid modest stipends. The year-long project occurred simultaneously in the first and second years allowing complete mapping of the preclinical curriculum in one calendar year. Identifying the content gaps was only the beginning, as it quickly became obvious that there was a need for instructional material to fill those gaps.

Early in the process, the faculty identified time as a major obstacle to develop curricula materials: time to research the topics and time in the curriculum to cover them. To address the time constraints, various curricular materials were developed including a PubMed search tool [5]. This tool facilitates literature searches through a sex and gender lens, thus allowing researchers easier access to sex- and gender-based content that is not categorized by familiar search terms. Subsequently, a slide library was developed that housed peer-reviewed lecture slides that could be easily inserted into an existing lecture. This material was developed to target undergraduate medical education. The Slide Library’s success centered on its ability to bypass the need for research time on the part of the course instructors who could now easily peruse slides that applied to their courses and insert then into existing lectures. Because the slide sets included speaking points, literature references and level of evidence, the instructors did not have to develop advanced expertise on the subject on their own. The cost of production was minimal as faculty and medical students volunteered time to research and produce the peer-reviewed slides as a scholarly activity.

Involvement of students and inter-professional faculty from the beginning of the process also led to the development of award-winning online interactive modules covering topics, such as osteoporosis, diabetes, cardiovascular disease, and others. Designed as brief, three-part interactive modules, the components can be threaded throughout a block, a course, or several years. Additionally, these modules can be used as stand-alone elements or as resources for flipped classrooms, where the module is assigned as homework and the class time can be reserved for discussion or other interactive activities. Development of these modules was much more time intensive, requiring 6 to 12 months to develop and produce in collaboration with instructional designers from Texas Tech University’s undergraduate campus. The cost of US$25,000 per module was covered by grants from the Laura W. Bush Institute as well as the Texas Tech University Health Science Center Schools of Medicine, Nursing, and Pharmacy.

Over time, the increase in student awareness of sex and gender as a biological variable that influences all aspects of health and disease led to faculty interest in expanding their own knowledge resulting in the development of the Online Continuing Medical Education and Certificate Program in Sex and Gender Specific Health. Under development with the support of the Laura W. Bush Institute for Women’s Health, the continuing professional development site (http://www.laurabushinstitute.org/cme/default.aspx) will offer a certificate program for physicians, nurses, pharmacists, and other interested health professionals.

The inter-professional approach cannot be overemphasized, as it led to incorporation of the content, initially targeted towards undergraduate medical education, into the School of Nursing and School of Pharmacy curricula after their own student audits. Both schools have provided financial support and led the development and production of online modules as well as other contents. More significantly, the Sex and Gender Based Medicine Curriculum and websites were changed to Sex and Gender Based Health to reflect the inter-professional nature of the content.

These major accomplishments at TTUHSC may be attributable to the approach to sex and gender, not as a women’s health issue, but a basic patient care issue. This approach contributed to early acceptance from faculty as well as students. Additionally, the establishment of the Sex and Gender Specific Health website (http://www.sexandgenderhealth.org) as an inter-professional central repository for all of the content provided a broader base of support through faculty and student awareness supporting accessibility and credibility to the discipline. The website also serves as the meeting place for the ongoing conversation regarding the contribution of sex and gender to personalized medicine.

## Conclusion

Each educational setting has unique challenges to overcome for innovation of the medical curriculum. However, common among them is the challenge to provide precise definitions of sex- and gender-based medicine and research in order to overcome unconscious bias so that the scientific evidence trumps political forces. Emphasis on improved patient care is critical to the conversation. Significant resources must be allocated to the initiative, and the initiative must be managed by persons with the vision and passion to meet the challenges. Engagement of the learner in the earliest stages of curricula development has proven to be a valuable resource in implementing change. In support, the European Medical Student Organization dedicated year 2016 to focus particularly on gender medicine (http://emsa-europe.eu/gender-medicine/). Although some of the programs specifically targeted faculty training in topics of sex and gender, many of the resources were developed to facilitate faculty involvement and reduce the time needed for a mentor to develop learning materials and expertise (databases, search engines, slide sets, etc.). Existing resources now can be adopted and adapted to many types of learners and learning environments. Those discussed within this article as well as those listed in Table 1 provide necessary frameworks and information upon which to build curricular concepts that can be embedded into existing paradigms.

**Table 1:** Additional resource material for use in the development of sex- and gender-medical curricula

Articles outlining experimental design methodology	Becker JB, Arnold AP, Berkley KJ, Blaustein JD, Eckel LA, Hampson E et al. Strategies and Methods for Research on Sex Differences in Brain and Behavior. Endocrinology. 2005;146:1650–73.
	Greenspan JD, Craft RM, LeResche L, Arendt-Nielsen L, Berkley KJ, Fillingim RB et al. Studying sex and gender differences in pain and analgesia: A consensus report. Pain. 2007;132:S26-S45.
	Miller VM, Kaplan JR, Schork NJ, Ouyang P, Berga SL, Wenger NK et al. Strategies and Methods to Study Sex Differences in Cardiovascular Structure and Function: A Guide for Basic Scientists. Biol Sex Differ. 2011;2:14. doi:10.1186/2042-6410-2-14.
	Shah K, McCormack CE, A BN. Do you know the sex of your cells? Am J Physiol Cell Physiol. 2014;306:C3-C18.
	Ritz SA, Antle DM, Cote J, et al. First steps for integrating sex and gender considerations into basic experimental biomedical research. FASEB journal: official publication of the Federation of American Societies for Experimental Biology 2014;28:4-13.
	Ouyang P, Wenger NK, Taylor D, et al. Strategies and methods to study female-specific cardiovascular health and disease: a guide for clinical scientists. Bio. Sex Differences 2016; 7:19. Doi:10.1186/s13293-016-0073-y
Textbooks	Einstein G. Sex and the Brain. MIT. 2007.
	Legato M. Principles of Gender-Specific Medicine. Elsevier. 2011. 2nd ed.
	Oertelt-Prigione, Regitz-Zagrosek. Clinical Aspects of Gender Specific Medicine. Springer. 2012.
	Schenk-Gustafsson K, DeCola PR, Pfaff SW, Plsetsky DS. Handbook of Clinical Gender Medicine. Karger.2012.
	Regitz-Zagrosek, V. ed. Sex and Gender Differences in Pharmacology. Springer-Verlag 2012.
	Mattison, DR. ed. Clinical Pharmacology during Pregnancy. Elsevier, 2013.
	Neigh G, Mitzelfelt, M. Sex Differences in Physiology. Academic Press Elsevier, 2016.

In spite of the successes exemplified here, unknown challenges in the future will be influenced by how the scientific and medical communities articulate, implement, and validate protocols to investigate sex and gender differences. The advent of the genomic era has made clear the ubiquity and magnitude of the variability of living things and the plasticity of the phenome, impacted as it is in all species by environment, age, and experience.

One challenge, difficult to dispel, is the idea that gender-specific medicine means women’s health only. This has kept the benefits of gender medicine from men, boys, and girls. Changing the name of most of the organizations currently advocating and developing gender-specific medicine to include “men and women” rather than just “women” in their group name would help dispel this notion. Thus, the consensus of this panel of experts in sex and gender curricula development is that the integration of sex and gender into the medical education will improve the education of future doctors, ultimately leading to improved medical care of all.
